# Source matters, AI anxiety less so: comparing employee reactions to human, AI, and hybrid performance feedback and the limited role of AI anxiety

**DOI:** 10.3389/fpsyg.2026.1924487

**Published:** 2026-08-17

**Authors:** Janka Laura Marót, Tamara Palcsó, Zsolt Péter Szabó

**Affiliations:** 1 Doctoral School of Psychology, ELTE Eötvös Loránd University, Budapest, Hungary; 2 Institute of Psychology, ELTE Eötvös Loránd University, Budapest, Hungary; 3 Institute of Strategy and Management, Corvinus University of Budapest, Budapest, Hungary

**Keywords:** AI anxiety, AI-generated feedback, disclosure effect, feedback source, generative artificial intelligence, human-AI collaboration, performance feedback

## Abstract

**Introduction:**

Large language models (LLMs) are increasingly integrated into human resource management practices and are becoming capable of supporting managerial tasks such as performance feedback. Although these technologies may improve the efficiency and quality of feedback delivery, it remains unclear whether knowledge of AI involvement influences recipients’ reactions to it.

**Methods:**

This work presents results from a vignette-based experiment (*N* = 192) examining employees’ reactions to a hypothetical performance feedback scenario. Participants were randomly assigned to one of four disclosed feedback-production workflows (fully human-written, fully AI-generated, AI-generated and human-refined, or human-written and AI-refined), while the feedback text itself was identical across conditions. Participants evaluated the feedback on a range of emotional, motivational, relational, and performance-related outcomes while AI anxiety was measured as a covariate in the analysis.

**Results:**

Results demonstrated that fully AI-generated feedback elicited less favorable affective, motivational, relational, and performance-related responses than fully human-written feedback, whereas hybrid feedback generally occupied an intermediate position, with human-written feedback refined by AI producing outcomes comparable to purely human feedback. AI anxiety showed only limited effects.

**Discussion:**

These findings suggest that the perceived source of performance feedback influences how feedback is evaluated, whereas AI anxiety showed only limited associations with employees’ reactions. The discussion addresses the theoretical implications of these findings for performance feedback, as well as their practical implications for organizations.

## 1 Introduction

Online discussions about generative artificial intelligence (genAI) increasingly reveal ambivalence toward managers’ use of large language models (LLMs) in employee feedback. Some employees worry that AI-generated feedback may undermine the interpersonal value of managerial communication. As one Reddit user put it, “I want to know what my manager thinks of my performance, not AI,” capturing the concern that feedback should reflect a manager’s own judgment rather than the output of an AI system. Others adopt a more instrumental view, arguing that AI is simply “a tool” that can help managers communicate more effectively. These contrasting reactions point to an emerging tension in organizational life: although employees may value feedback as a relational and evaluative signal from their manager, LLMs are increasingly capable of producing and revising human-like feedback messages (García Peñalvo and Vázquez Ingelmo, 2023; Meyer et al., 2024), and AI-based tools are becoming more common in managerial and human resource practices (Budhwar et al., 2023; Kellogg et al., 2020; Tambe et al., 2019).

This tension is further complicated by the fact that recipients usually see only the final feedback message, not the process through which it was produced. Managers may use LLMs to generate feedback, revise their own feedback, or refine AI-generated drafts, but this involvement may not be visible in the final text.

Against this backdrop, the present study examines whether explicit source disclosure changes employees’ responses to otherwise identical feedback in a hypothetical workplace scenario. Specifically, through a vignette experiment, we examine a wide range of reactions including recipients’ emotional (affective response and rumination), motivational (willingness to correct mistakes and work motivation), relational responses (relationship with-, seeking help from-, and psychological distance from the supervisor) and perceived performance- and organization-related (expected performance and organizational commitment) reactions to feedback across different source conditions. In addition, we examine the potential role of AI anxiety as a covariate.

### 1.1 Literature review

Effective workplace feedback is important but difficult because it requires managers to combine accurate performance information with relationally sensitive communication. Although feedback can guide future behavior, clarify performance expectations, and support work motivation (Ilgen et al., 1979), it does not necessarily lead to performance improvement and may even undermine performance (Kluger and DeNisi, 1996). One reason for these mixed effects is the cognitive and relational complexity of feedback delivery (London, 2003). Feedback effectiveness depends not only on the content of the message, but also on the supervisor-subordinate relationship, recipients’ perceptions and reactions, goal-setting processes, and the way feedback is delivered (Heine et al., 2026; Lechermeier and Fassnacht, 2018; Sleiman et al., 2020).

When these factors are poorly managed, feedback may elicit negative affective responses, which can even facilitate learning under certain conditions (Zhao, 2011), but they also lead to increased turnover intention, increased counterproductive work behavior, defensiveness, anger, interpersonal conflict, and disengagement rather than development (Bakker and Leiter, 2010; Belschak and Den Hartog, 2009; Besieux, 2017; Marót et al., 2024). Negative feedback experiences may weaken perceptions of supervisory support and relationship quality (Heine et al., 2026). Consequently, employees may become less willing to seek guidance or assistance from their supervisor (Berkovich and Eyal, 2018). Negative feedback experiences may also trigger rumination, causing employees to repeatedly think about the feedback after the interaction (Mendiratta and Singh, 2026). Together, these affective, cognitive, and relational reactions may ultimately influence broader work-related attitudes and outcomes, including organizational commitment (Belschak and Den Hartog, 2009). These potential consequences illustrate both the importance and the complexity of effective feedback delivery, creating a potential role for AI-supported feedback tools, particularly LLMs, while also raising questions about how such tools may affect the meaning and interpretation of managerial feedback.

Artificial intelligence-supported feedback tools may help managers address some of the cognitive and linguistic demands of feedback delivery. Research on algorithmic judgment suggests that people often perceive algorithmic recommendations as useful and reliable (Logg et al., 2019), and AI systems may improve assessment quality in well-structured tasks by making evaluations more accurate, consistent, fair, or potentially less biased than those of human managers (Tong et al., 2021; Qin et al., 2023). Given the cognitive constraints and potential biases that often limit supervisors, AI may also support the generation of more personalized and comprehensive feedback (Tong et al., 2021). Thus, from a practical perspective, LLMs may assist managers by helping them draft, structure, or refine feedback messages.

At the same time, AI-supported feedback may be problematic because managerial feedback is not merely informational, but also relational. As discussed above, feedback communicates not only what an employee did well or poorly, but also whether the manager paid attention, exercised judgment, took responsibility for the employee’s development, was available, and showed support. For this reason, feedback generated or substantially shaped by AI may be interpreted differently from feedback written by a human manager, even when the content is similar. Consistent with this view, although employees accept AI-generated feedback when it is perceived as clear, useful, and accurate, it also creates greater psychological distance because employees do not perceive AI systems as social actors, which may weaken its relational impact (Hein et al., 2024). Prior research suggests that disclosing AI or algorithmic involvement can reduce trust, perceived credibility, fairness, and warmth, even when the quality of the output is comparable to human judgment (Nazaretsky et al., 2026; Schilke and Reimann, 2025; Tong et al., 2021). Thus, source disclosure may change how recipients evaluate otherwise identical feedback. This concern is especially relevant in contexts that require intuition, contextual understanding, or subjective judgment, where AI-based evaluations may be perceived as less fair and less trustworthy than human evaluations (Lee, 2018). Performance feedback is such a context, because recipients may expect the message to reflect the manager’s own knowledge, judgment, and involvement.

Disclosure effects may also depend on individual differences in how people perceive and respond to AI. People vary in their trust in AI systems and in their comfort with AI involvement in human decision-making (Park et al., 2024). This may be especially relevant in managerial feedback, where AI involvement can raise concerns about human judgment, responsibility, or care. One relevant individual difference is AI anxiety, which refers to apprehension or discomfort associated with AI technologies. Because higher AI anxiety is associated with more negative attitudes toward AI (Kaya et al., 2024), individuals higher in AI anxiety may react more negatively when feedback is explicitly presented as AI-generated or AI-supported.

These concerns do not mean that AI-supported feedback must be fully automated. In practice, managers may use AI in more limited ways, for example to draft or refine feedback while retaining responsibility for the final message. Such hybrid forms of feedback may preserve some advantages of AI, including efficiency and linguistic support, while maintaining human involvement in the evaluation process (Biswas et al., 2024; Jarrahi, 2018; Trunk et al., 2020). Similar approaches have shown promise in educational settings, where human–AI collaboration may reduce some of the negative reactions associated with fully automated feedback (Henderson et al., 2026; Tossell et al., 2024; Zawiah et al., 2023; Zhang et al., 2025). However, less is known about how hybrid feedback is perceived in workplace feedback contexts.

In sum, AI-supported feedback creates a dilemma for managerial feedback. AI may help managers produce feedback that is more consistent, comprehensive, and useful, but its involvement may also change how feedback is interpreted, especially when recipients expect performance feedback to reflect the manager’s own judgment and involvement. Therefore, understanding AI-supported feedback requires exploring how the disclosed source of feedback influences employees’ evaluations of it.

### 1.2 The present research

The preceding arguments suggest that AI-supported feedback should be examined in terms of how AI is involved in feedback production. We therefore distinguish between four feedback-production workflows: fully human-written feedback (H), fully AI-generated feedback (AI), AI-generated feedback refined by a human (AIxH), and human-written feedback refined by AI (HxAI).

Although AI-generated feedback may offer some advantages over human-written feedback, feedback is also a relational act, therefore, feedback disclosed as fully human-written should elicit more positive reactions than feedback disclosed as fully AI-generated.

H1: Fully human-written feedback will elicit more positive emotional, motivational, and perceived performance-related reactions than fully AI-generated feedback.

Hybrid feedback may occupy an intermediate position. Because such feedback involves AI, it may be evaluated less positively than fully human-written feedback. However, because it also involves human input, it may be evaluated more positively than fully AI-generated feedback. Because the relative evaluation of hybrid feedback in managerial feedback contexts remains unclear, we examined this question in an exploratory manner.

Exploratory RQ1: How do recipients respond to hybrid human–AI feedback relative to fully human-written and fully AI-generated feedback?

At the same time, the two hybrid workflows may not be psychologically equivalent. AI-generated feedback refined by a human may signal that the human manager had final oversight, whereas human-written feedback refined by AI may signal that the manager originated the evaluation but used AI to improve the wording. Because these workflows imply different forms of human and AI involvement, we examined their difference as a research question.

Exploratory RQ2: Do recipients respond differently to AI-generated feedback refined by a human compared with human-written feedback refined by AI?

Finally, reactions to AI involvement may depend on individual differences. In particular, individuals higher in AI anxiety may respond more negatively when feedback is presented as AI-generated or AI-supported. Because individual differences in attitudes toward AI may influence evaluations of AI-supported feedback, AI anxiety was measured and included as a covariate in the analyses. Although the role of AI anxiety in managerial feedback contexts remains insufficiently understood, its potential influence was considered exploratory.

Exploratory RQ3: Do differences in recipients’ reactions to disclosed feedback sources remain when accounting for AI anxiety?

To address these questions, we conducted an experimental vignette study in which participants were presented with an identical feedback text embedded in a hypothetical workplace scenario, varying only in the disclosed source (human, AI, and hybrid workflows). By holding the feedback message constant, this experimental vignette design allowed us to isolate the effect of source disclosure on participants’ immediate reactions. We examined a wide range of outcomes, including recipients’ emotional (affective response and rumination), motivational (willingness to correct mistakes and work motivation), relational (relationship with-, seeking help from-, and psychological distance from the supervisor), and performance- and organization-related reactions (expected performance and organizational commitment). In addition, we explored the potential role of AI anxiety as a covariate. Although this design supports causal inference about immediate scenario-based reactions, it captures anticipated responses to a standardized hypothetical situation rather than employees’ actual behavior or longer-term reactions to consequential feedback within an ongoing workplace relationship.

## 2 Materials and methods

Data collection was conducted in 2025 through an online Qualtrics survey. Participants provided informed consent at the beginning in accordance with the Declaration of Helsinki. Those who did not provide consent were directed to the end of the survey and did not participate further. The study received ethical approval from the Research Ethics Committee of Corvinus University of Budapest (file number: KRH/256/2025).

Statistical analysis and data visualization were conducted in R version 4.6.1 (R Core Team, 2026). The tidyverse (Wickham et al., 2019), ggplot2 (Wickham, 2016), and sjPlot (Lüdecke, 2025) packages were used for data transformation, analysis, and visualization. Power analysis was conducted using G*Power 3.1.9.7 (Faul et al., 2009).

### 2.1 Participants

An *a priori* power analysis indicated that, assuming an expected effect size of *f* = 0.25 (power = 0.80, alpha = 0.05), the required sample size was 179 participants. A total of 230 Hungarian participants were recruited through convenience sampling via social media platforms and the researchers’ professional and personal networks. Participants were excluded if they did not provide informed consent (*n* = 0), or were currently unemployed (*n* = 18). To minimize low-quality responses, we included one attention check and excluded 20 participants who failed it. The final sample consisted of 192 employees, which met the sample size requirement indicated by the *a priori* power analysis. A step-by-step breakdown of participant counts and exclusions across conditions is provided in Supplementary Material 1.

Of the final sample, 105 participants were female (54.69%) and 87 were male (45.31%). Participants ranged in age from 19 to 65 years (*M*_*age*_ = 32.34 years, *SD*_*age*_ = 11.84 years). Regarding education, 130 participants had a higher education degree (67.71%), 58 had completed high school or lower education (30.21%), and four selected “other” (2.08%). In terms of organizational position, 127 participants worked at the subordinate level (66.15%), 32 were middle managers (16.67%), 12 were executives (6.25%), and 21 selected “other” (10.94%).

### 2.2 Procedure

Participants first completed self-reported questions on work status and basic demographic characteristics. They were then presented with a vignette describing a hypothetical workplace situation in which they were asked to imagine receiving performance feedback from a fictional supervisor. Participants were randomly assigned to one of four conditions that differed only in the disclosed feedback-production process: the supervisor was described as either personally writing all feedback (fully human-written, *n* = 42), using AI-generated feedback without modification (fully AI-generated, *n* = 56), reviewing and approving AI-generated feedback before sending it (AI-generated refined by human, *n* = 44), or personally writing feedback and subsequently refining it with the assistance of AI (human-written refined by AI, *n* = 50). After this, all participants were presented with the same “excerpt […] from their most recent performance review.” The feedback contained both positive (“strong in meeting deadlines and completing administrative tasks”) and developmental elements (“pay more conscious attention to joint discussions and communication”). Participants subsequently completed measures assessing their reactions to the feedback. The full vignettes are presented in Supplementary Material 2.

### 2.3 Measures

Immediately after the vignette, participants answered three open-ended questions designed to capture their initial thoughts, feelings, and intended actions in response to the feedback. They were asked to describe in one or two sentences what thoughts came to mind when they received the feedback, how they felt about the feedback, and how they would act after receiving it.

Participants then completed a manipulation check to assess whether they correctly understood the disclosed source of the feedback. They answered the question, “According to the situation, How does your manager provide feedback?” Response options were: “personally themselves,” “using artificial intelligence, without modification,” “using artificial intelligence, with personal review and approval,” and “personally, then refined and corrected using artificial intelligence.”

The next block consisted of the main reaction measures. Self-reported single-item outcomes were rated on 7-point Likert-type scales. The assessed outcomes were general affective response (“How would that feedback make you feel?”; 1 = Very bad, 7 = Very good), rumination (“How much would you ruminate on this feedback throughout the day?”; 1 = Not at all, 7 = All the time), willingness to correct the mistake (“After this feedback, To what extent do you intend to correct the mistake?”; 1 = I would not intend to at all, 7 = I would fully intend to), openness to ask for help from the supervisor (“After this feedback, How likely do you think it is that you would go to your supervisor with professional questions?”; 1 = Not likely at all, 7 = Entirely likely), organizational commitment (“How would this feedback affect your commitment to the organization?”; 1 = It would greatly reduce it, 7 = It would greatly increase it), relationship with the supervisor (“How would this feedback affect the relationship with your supervisor?”; 1 = It would greatly worsen it, 7 = It would greatly improve it), expected performance (“How would this feedback affect your performance?”; 1 = It would greatly worsen it, 7 = It would greatly improve it), and work motivation (“How would this feedback affect your work motivation?”; 1 = It would greatly reduce it, 7 = It would greatly increase it). Perceived psychological distance from the feedback source was assessed with a four-item scale based on the theoretical framework of Hein et al. (2024). An example item is: “I feel that the person giving feedback is emotionally close to me.” Items were rated on a 5-point Likert-type scale ranging from 1 = not at all to 5 = completely. The scale showed high internal consistency (α = 0.89).

Finally, AI anxiety was assessed as an exploratory measure using the AI Anxiety Scale (AIAS; Wang and Wang, 2022). Because no validated Hungarian version was available, the items were translated using the back-translation method (Brislin, 1970). The scale includes 21 items rated on a 7-point Likert-type scale ranging from 1 = not at all to 7 = completely and covers four dimensions: learning anxiety (8 items; e.g., “I feel anxious when I have to learn how to use AI technologies/products”), job replacement anxiety (6 items; e.g., “I am afraid that AI technologies/products will replace humans”), sociotechnical blindness (4 items; e.g., “I am afraid that AI technology/products could be misused”) and AI configuration anxiety [3 items; e.g., “I find humanoid AI technologies/products (e.g., humanoid robots) frightening.”]. Although the AIAS comprises multiple dimensions, it was developed to capture a broader AI anxiety construct. Accordingly, subsequent analyses were conducted using the overall AIAS score rather than the individual subscale scores (Wang and Wang, 2022). Cronbach’s alpha coefficients indicated excellent reliability for the total scale (α = 0.94)

### 2.4 Data analysis

To address the study objectives, a manipulation check was conducted using a chi-square test to examine whether participants correctly recognized the disclosed source of the feedback. Differences in reactions to feedback across the four source conditions were examined using multivariate analysis of variance (MANOVA). MANOVA was used instead of a series of separate ANOVAs because it accounts for potential correlations among the outcome variables (Grice and Iwasaki, 2007). AI anxiety was included in the model as a covariate to test whether reactions to different feedback sources varied with individual differences in AI anxiety. *Post hoc* pairwise comparisons of estimated marginal means (EMMs) were conducted using Bonferroni-adjusted *p*-values.

## 3 Results

### 3.1 Preliminary analysis

The manipulation check confirmed effectiveness: a chi-square test indicated a significant association between the disclosed and perceived source of feedback, χ^2^(9) = 162.28, *p* < 0.001, suggesting that participants were able to correctly identify (*n* = 123, 64.06%) the feedback source above chance level (25%).

Pre-manipulation differences in sociodemographic variables were examined to assess the random allocation of participants to conditions. The analysis revealed no statistically significant baseline differences between the four conditions for gender (*p* = 0.808) and position (*p* = 0.421), except for level of education (*p* = 0.029). Therefore, all analyses were verified by including education level as a control variable, which did not yield any meaningful changes in the outcomes.

### 3.2 Main analyses

A multivariate analysis of variance (MANOVA) was conducted to test whether the disclosed source of the feedback (human, AI, AI-generated refined by a human, and human-written refined by AI) influenced participants’ reactions across nine outcome variables: willingness to correct the mistake, openness to ask for help from the supervisor, organizational commitment, relationship with the supervisor, expected performance, general affective response, rumination, motivation, and perceived psychological distance. Using Pillai’s trace, the overall multivariate effect of feedback source was significant, *V* = 0.37, *F*(27, 546) = 2.86, *p* < 0.001.

Follow-up univariate ANOVAs revealed significant, mostly small-to-moderate effects of feedback source on affective response, *F*(3, 188) = 5.16, *p* = 0.002, η^2^ = 0.08; willingness to correct the mistake, *F*(3, 188) = 10.45, *p* < 0.001, η^2^ = 0.14; openness to ask for help from the supervisor, *F*(3, 188) = 13.17, *p* < 0.001, η^2^ = 0.17; organizational commitment, *F*(3, 188) = 11.67, *p* < 0.001, η^2^ = 0.16; relationship with the supervisor, *F*(3, 188) = 16.81, *p* < 0.001, η^2^ = 0.21; expected performance, *F*(3, 188) = 6.70, *p* < 0.001, η^2^ = 0.10; motivation, *F*(3, 188) = 9.43, *p* < 0.001, η^2^ = 0.13; and perceived psychological distance, *F*(3, 188) = 20.12, *p* < 0.001, η^2^ = 0.24. No significant effect was found for rumination, *F*(3, 188) = 0.57, *p* = 0.64, η^2^ = 0.01.

**FIGURE 1:**
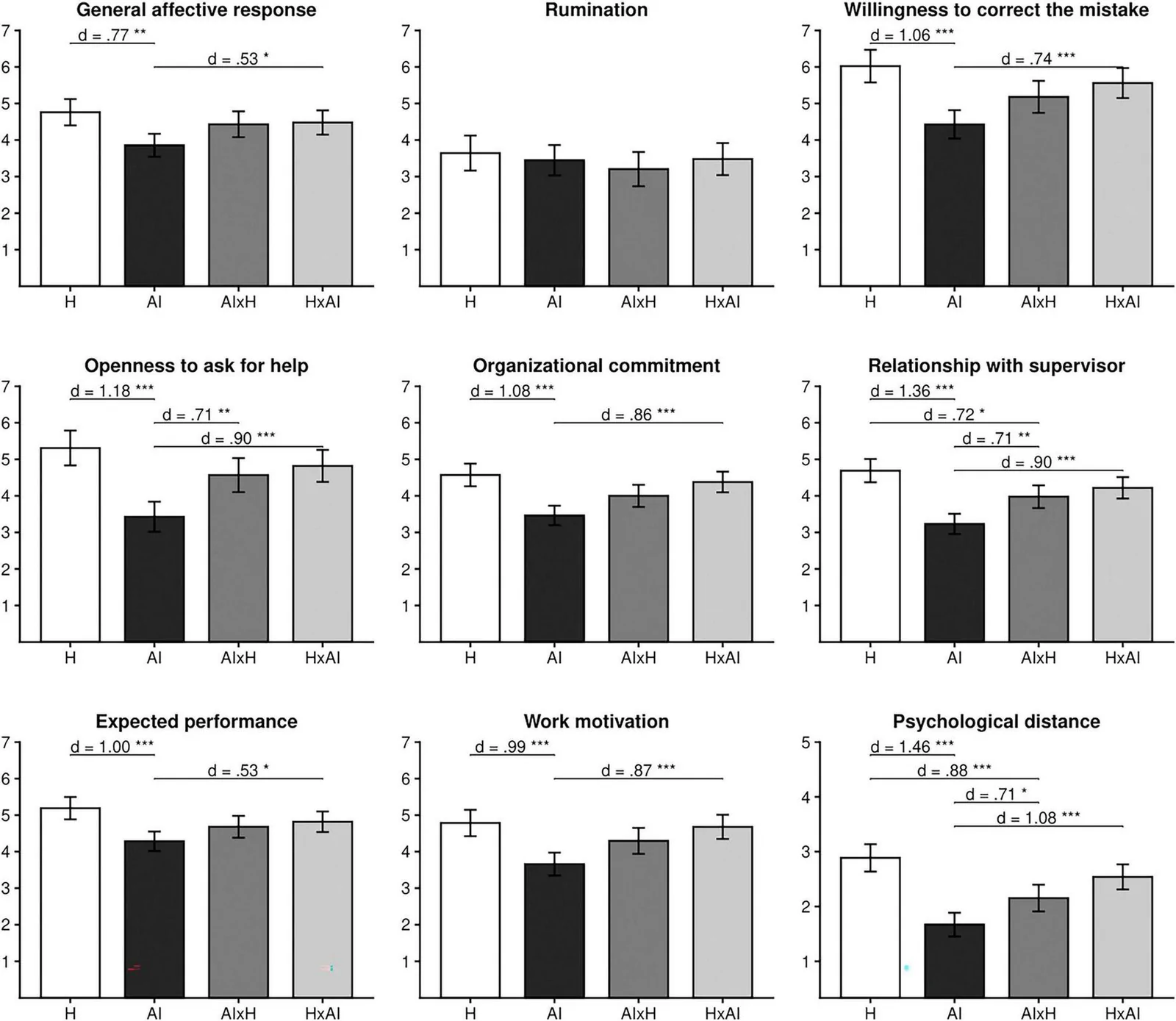
Effects of disclosed source on outcome variables. Error bars represent the lower and upper bounds of 95% confidence intervals. Single-items were ranged between 1 and 7. For psychological distance (ranged between 1 and 5), higher values indicate a smaller perceived distance. Non-significant pairwise comparisons are not displayed for clarity. *p < 0.05, **p < 0.01, ***p < 0.001.

Bonferroni-adjusted pairwise comparisons showed that fully AI-generated feedback was evaluated less favorably than fully human feedback on affective response (EMMs = 3.86 vs. 4.76, *p* = 0.002), willingness to correct the mistake (EMMs = 4.43 vs. 6.02, *p* < 0.001), openness to ask for help (EMMs = 3.43 vs. 5.31, *p* < 0.001), organizational commitment (EMMs = 3.46 vs. 4.57, *p* < 0.001), relationship with the supervisor (EMMs = 3.23 vs. 4.69, *p* < 0.001), expected performance (EMMs = 4.29 vs. 5.19, *p* < 0.001), motivation (EMMs = 3.66 vs. 4.79, *p* < 0.001), and perceived psychological distance (EMMs = 1.67 vs. 2.89, *p* < 0.001). Fully AI-generated feedback was also evaluated less favorably than human-written feedback refined by AI on affective response (EMMs = 3.86 vs. 4.48, *p* = 0.046), willingness to correct the mistake (EMMs = 4.43 vs. 5.56, *p* = 0.001), openness to ask for help (EMMs = 3.43 vs. 4.82, *p* < 0.001), organizational commitment (EMMs = 3.46 vs. 4.38, *p* < 0.001), relationship with the supervisor (EMMs = 3.23 vs. 4.22, *p* < 0.001), expected performance (EMMs = 4.29 vs. 4.82, *p* = 0.042), motivation (EMMs = 3.66 vs. 4.68, *p* < 0.001), and perceived psychological distance (EMMs = 1.67 vs. 2.54, *p* < 0.001). When compared to AI-generated feedback refined by a human, fully AI-generated feedback was again associated less favorably on openness to ask for help (EMMs = 3.43 vs. 4.57, *p* = 0.002), relationship with the supervisor (EMMs = 3.23 vs. 3.98, *p* = 0.003), and perceived psychological distance (EMMs = 1.67 vs. 2.15, *p* = 0.023). No other pairwise differences were significant. Figure 1 depicts these differences, and Table 1 summarizes them (see more details in Supplementary Materials 3, 4).

**TABLE 1:** Summary of the main analysis results.

Outcome	*EMM* (H)	*EMM* (AI)	*EMM* (AIxH)	*EMM* (HxAI)	*F* (3, 188)	*p*	η^2^
Affective response	4.76	3.86	4.43	4.48	5.16	0.002	0.08
Rumination	3.64	3.45	3.20	3.48	0.57	0.640	0.01
Willingness to correct the mistake	6.02	4.43	5.18	5.56	10.45	<0.001	0.14
Openness to ask for help	5.31	3.43	4.57	4.82	13.17	<0.001	0.17
Organizational commitment	4.57	3.46	4.00	4.38	11.67	<0.001	0.16
Relationship with the supervisor	4.69	3.23	3.98	4.22	16.81	<0.001	0.21
Expected performance	5.19	4.29	4.68	4.82	6.70	<0.001	0.10
Work motivation	4.79	3.66	4.30	4.68	9.43	<0.001	0.13
Psychological distance	2.89	1.67	2.15	2.54	20.12	<0.001	0.24

H = fully human-written, AI = fully AI-generated, AIxH = AI-generated and refined by a human, and HxAI = human-written and refined by AI. Higher scores indicate more positive reactions, except for rumination. For psychological distance, higher scores indicate lower perceived distance. η^2^ = eta squared.

### 3.3 Robustness analysis among participants who passed the manipulation check

To examine the robustness of the findings, the main analyses were repeated among participants who correctly identified the disclosed feedback source in the manipulation check (*n* = 123). The multivariate effect of feedback source remained significant, *V* = 0.70, *F*(27, 339) = 3.83, *p* < 0.001. The univariate results followed the same overall pattern as those obtained in the full sample. Significant effects emerged for all outcomes except rumination, and the effect sizes were generally larger, ranging from η^2^ = 0.19 to 0.42. The main pairwise differences were also replicated, although several additional significant comparisons emerged. Overall, the robustness analysis supported the main pattern of findings while indicating more pronounced differences among participants who correctly identified the disclosed source. Detailed results are presented in Supplementary Material 5.

### 3.4 AI anxiety as a covariate

Artificial intelligence anxiety was assessed after the outcome measures to avoid drawing participants’ attention to AI-related concerns before they evaluated the feedback. Given this measurement order, condition assignment may have influenced participants’ reported AI anxiety. Before conducting the covariate analysis, we therefore examined whether AIAS scores differed across conditions. No significant difference was found, *F*(3, 188) = 0.55, *p* = 0.651.

Artificial intelligence anxiety was subsequently included as a covariate in the model examining its association with participants’ reactions to feedback. Using Pillai’s trace, the multivariate effect of feedback source remained significant, *V* = 0.37, *F*(27, 543) = 2.85, *p* < 0.001 (see more details in Supplementary Material 6). In contrast, the multivariate effect of AI anxiety was not significant, *V* = 0.08, *F*(9, 179) = 1.73, *p* = 0.085.

At the univariate level, AI anxiety was significantly associated only with rumination, *F*(1, 187) = 14.76, *p* < 0.001, ηp^2^ = 0.07, indicating that participants reporting higher AI anxiety also reported greater rumination following feedback. No other significant effects of AI anxiety emerged for outcomes (all *ps* > 0.301, see more details in Supplementary Material 7).

Taken together, these results suggest that although AI anxiety was associated with greater rumination overall, it did not systematically alter the pattern of reactions to the different feedback sources.

## 4 Discussion

Building on the growing body of research examining the integration of AI into performance appraisal processes (Biswas et al., 2024; Jarrahi, 2018; Pei et al., 2024; Tong et al., 2021), the present study aimed to investigate how the knowledge of the feedback source influences reactions to feedback in an organizational context. Specifically, we compared how employees react to feedback when the source (fully human-written, fully AI-generated, AI-generated refined by a human, and human-written refined by AI) is explicitly revealed. In addition, we explored AI anxiety as a covariate in the relationship between disclosed feedback sources and reactions.

Consistent with our hypotheses and prior research (Nazaretsky et al., 2026; Tong et al., 2021), fully human-written feedback elicited more favorable reactions than fully AI-generated feedback (H1). Specifically, human feedback elicits more positive affective responses, greater willingness to correct mistakes, higher openness to ask for help, stronger organizational commitment, better relationship with supervisors, higher motivation, and lower perceived psychological distance. These findings support the disclosure effect: once the source of feedback is known, it alters its effect (Tong et al., 2021).

These findings also extend the broader workplace feedback literature by demonstrating that recipients’ reactions depend not only on the content of the message but also on how the feedback is delivered and who is perceived to be responsible for it (Ilgen et al., 1979; London, 2003; Lechermeier and Fassnacht, 2018). Although participants received identical feedback, source disclosure influenced emotional, motivational, organizational, and relational outcomes. Differences in openness to ask for help, relationship with the supervisor, and psychological distance align with research emphasizing that feedback is embedded in the supervisor-employee relationship and may shape employees’ willingness to seek subsequent guidance and support (Berkovich and Eyal, 2018; Heine et al., 2026).

One possible explanation for this pattern may lie in perceptions of source credibility. Prior research suggests that feedback can be effective only in influencing behavior if it is accepted and internalized by the recipient (Ashford, 1986). Feedback acceptance is closely related to the extent to which the receiver perceives the source as credible and professionally competent, as feedback from credible sources has been shown to increase acceptance and reduce defensiveness (Ilgen et al., 1979). Regarding supervisors, credibility has been shown to not only enhance feedback acceptance, but motivation, and ultimately performance as well, partly because it signals trust and reflects the quality of the supervisor-subordinate relationship (Son and Kim, 2016; Steelman and Rutkowski, 2004; van de Ridder et al., 2015).

From this perspective, the negative reactions toward disclosed AI involvement may reflect not the content of the feedback itself, but changes in how the source is perceived. In human interactions, a credible source is perceived as someone who has knowledge about the recipient (expertise), is willing to share their knowledge with the recipient (trustworthiness), and cares about the recipient (goodwill) (Perloff, 2003). While AI systems may fulfill the expertise dimension due to their technical advantages, their ability to convey trustworthiness and goodwill remains questionable. On the one hand, AI systems are designed to generate responses based on their “best available knowledge,” even if this means hallucinations can occur (Kaate et al., 2025). On the other hand, it is not clear whether AI should be designed to simulate relational qualities such as trustworthiness and goodwill, as doing so may make users vulnerable to its influence (Carroll, 2025, Henderson et al., 2026). When supervisors incorporate AI into the feedback process, the boundaries of authorship and responsibility may become blurred, potentially making the credibility of the feedback source more ambiguous. As a result, it becomes a key question how to design AI-supported feedback that preserves source credibility and delivers effective feedback.

Our study also reinforces prior research on perceptions of human–AI collaboration (Biswas et al., 2024; Jarrahi, 2018; Trunk et al., 2020), suggesting that hybrid feedback may offer a promising solution. Although the mean values indicate a clear pattern in which purely human feedback is evaluated most positively, followed by human-written feedback refined by AI, then AI-generated feedback refined by human, and finally fully AI-generated feedback, statistical analyses reveal a more nuanced picture. Addressing RQ1, human-written feedback refined by AI did not differ significantly from purely human feedback and was evaluated more positively than fully AI-generated feedback on all outcomes except rumination. AI-generated feedback refined by a human also outperformed fully AI-generated feedback, although these differences were limited to relational dimensions, including openness to ask for help, relationship quality with the supervisor, and psychological distance. At the same time, addressing RQ2, no significant differences emerged between the two hybrid workflows, suggesting that recipients do not meaningfully distinguish between different forms of human–AI collaboration despite their theoretically distinct patterns of human and AI involvement. Interpreted through the source credibility perspective outlined above, these findings may indicate that hybrid forms of feedback evoke source-related perceptions similar to those associated with purely human feedback. Importantly, the few remaining differences emerged between purely human feedback and AI-generated feedback refined by a human, and were concentrated in relational dimensions, suggesting that AI involvement may primarily affect the interpersonal component of the feedback process even when overall evaluations remain largely similar. This pattern is consistent with the possibility that preserving human authorship while using AI primarily as a refinement tool may be less disruptive to relationship-related perceptions than workflows in which AI is perceived as the originator of the feedback, while still allowing managers to draw on the broader potential of AI to improve consistency, comprehensiveness, and linguistic quality (Qin et al., 2023; Tong et al., 2021). Regarding our research question about how individual differences influence reactions to disclosed AI involvement in feedback processes, AI anxiety did not appear to shape reactions to feedback in the present study (RQ3). However, it is possible that other AI-related variables, such as broader attitudes toward AI or technology, may play a more important role. In line with the source credibility perspective discussed above, it is also possible that reactions to AI-supported feedback are linked less to AI itself and more to how AI involvement influences perceptions of the feedback source and the relational context surrounding feedback delivery.

### 4.1 Contributions

Beyond comparing AI and human feedback, our study contributes to the evaluation of hybrid feedback categories, extending prior evidence from educational settings (Henderson et al., 2026; Tossell et al., 2024; Zawiah et al., 2023; Zhang et al., 2025) to organizational contexts, showing that - except for relational dimensions - they do not perform worse than human feedback. Furthermore, our findings extend the literature on factors (such as attitudes towards AI - Park et al., 2024 or anthropocentrism - Wang et al., 2026) shaping perceptions of AI use in performance appraisal by examining the role of AI anxiety.

From a practical perspective, our findings reinforce Wang et al. (2026) argument that fully replacing managers with AI, specifically in the context of feedback delivery, is not advisable. However, the hybrid approaches, particularly using AI as a reviewing or support tool, appear to be a viable option for improving both the quality and efficiency of performance appraisal processes. AI can help managers keep track of the many criteria outlined in prior studies (Heine et al., 2026; Lechermeier and Fassnacht, 2018; Sleiman et al., 2020), process large volumes of performance-related data, and provide data-driven insights to support more informed evaluations, while also assisting in the refinement of written feedback and improving its constructiveness. Implementing AI-supported feedback systems may also contribute to the long-term development of managers’ feedback-giving skills, as they receive continuous (constructive) input on how to improve their feedback. However, these potential effects need further investigation, as they were not examined in the present study.

### 4.2 Limitations and future research directions

Although the results are clear, this study has several limitations. First, our hypothetical vignette design cannot fully capture the complexity of real workplace dynamics. Although holding the text constant strengthens causal identification of the labeling effect, participants reported anticipated reactions to a salient disclosure rather than behavior following feedback from an actual supervisor. The large differences observed for some comparisons (up to *d* = 1.46) may therefore partly reflect label salience or demand characteristics and may overstate effects likely in field settings. Therefore, future research could benefit from examining feedback processes in more naturalistic, real-world settings involving actual managers and employees, while carefully considering ethical constraints.

Second, the use of single-item measures limits the precision of construct measurement. Therefore, our findings primarily reflect immediate reactions to feedback rather than deeper psychological constructs or long-term outcomes such as future performance. Future studies could address this limitation by employing validated multi-item scales to capture psychological constructs more reliably.

Third, given that the strongest effects were observed in relational dimensions (e.g., openness to ask for help, relationship with the supervisor, psychological distance), future research should further explore these aspects, as prior work highlights the central role of relational context in determining feedback effectiveness (Heine et al., 2026). In the present study, we focused primarily on employees’ reactions to feedback rather than on how they directly perceived the feedback source itself. Nevertheless, perceptions related to source credibility may represent an important underlying mechanism shaping responses to AI-supported feedback. Although prior research has already examined the credibility of AI-supported or AI-generated communication (e.g., Khan and Mishra, 2024; Ou et al., 2026; Shin, 2022), these studies have primarily been conducted outside workplace feedback contexts. Future studies could therefore benefit from directly examining how employees evaluate the credibility and perceived responsibility of AI-involved feedback sources in organizational contexts. Longitudinal research would be particularly valuable to examine how these perceptions evolve over time and influence longer-term outcomes.

Fourth, all participants were recruited in Hungary through convenience sampling. This non-probability approach creates self-selection concerns: people reached through the researchers’ networks or motivated to complete a feedback-related survey may differ systematically from the broader employee population. Also, the cultural differences in attitudes toward artificial intelligence may influence responses to AI-generated feedback. Prior research in the Hungarian context has shown that the majority of individuals hold ambivalent attitudes toward AI, simultaneously recognizing its potential benefits and risks, while only a minority can be characterized as clearly positive or clearly negative toward the technology (Horváth and Molnár, 2026). Consequently, reactions to AI-generated feedback observed in the present studies may partly reflect the specific attitudinal profile of Hungarian employees and may differ in populations with higher levels of AI acceptance or skepticism. In addition, due to the relatively small sample size, smaller effect sizes may not have been detected. Replication in larger and more demographically and culturally diverse samples is therefore needed.

Finally, two measurement limitations should be considered. First, the manipulation check assessed whether participants could correctly report the disclosed feedback-production workflow, but not whether they believed that the feedback had actually been produced in the stated way. Participants’ perceptions of the credibility of the disclosure may therefore have influenced their responses. Future studies should include an additional question assessing whether participants believed the disclosed source information. Second, AI anxiety was measured after the manipulation. Although AIAS scores did not differ significantly across conditions in the present sample, it remains unclear whether the observed pattern would have been the same if AI anxiety had been measured earlier. Future studies should therefore assess AI anxiety in a separate measurement wave so that participants are less likely to associate the scale with the purpose of the study. Future research should also explore additional moderating and mediating factors, such as attitudes toward AI or personality traits, that may shape responses to AI-supported feedback. It would also be valuable to examine how results change when the source of feedback is not “widely known” but only “assumed” by employees. In the present study, the disclosed feedback source was clearly defined, however, in real organizational contexts, transparency around feedback processes may be limited (Reddy Yanamala, 2023).

## 5 Conclusion

This study reinforces the disclosure effect: when the source of feedback is known, human feedback is preferred over AI-generated feedback. Importantly, no significant differences were found between human-written and human-written-AI-refined hybrid feedback, suggesting that appropriate human-AI collaboration represents an effective alternative in performance appraisal contexts, enabling more efficient feedback processes while maintaining quality. Altogether, these results suggest that organizations and managers should not view AI as a replacement for human judgment, but rather as a complementary tool that can enhance feedback processes when carefully integrated and appropriately managed.

## Data Availability

The datasets presented in this study can be found in online repositories. The names of the repository/repositories and accession number(s) can be found below: Open Science Framework: https://osf.io/whu35/overview?view_only=aec95000 601a4c5b8c3b81bd326683fd.
