# Supplementary material for: Source matters, AI anxiety less so: comparing employee reactions to human, AI, and hybrid performance feedback and the limited role of AI anxiety
Source: Front Psychol. 2026 Aug 17;17:1924487. doi: 10.3389/fpsyg.2026.1924487 (PMC13526566; doi:10.3389/fpsyg.2026.1924487)
Supplement: Supplementary file 1 [file Table_1.docx]

***Supplementary Materials***

**Supplementary Material 1.** Step-by-Step Participant Counts and Exclusions Across Experimental Conditions

**Supplementary Material 2.** Vignettes Used in the Study

**Supplementary Material 3.** Differences Between Conditions

**Supplementary Material 4.** Differences Between Conditions controlling for education

**Supplementary Material 5.** Robustness Analysis Among Participants Who Passed the Manipulation Check

**Supplementary Material 6.** Differences Between Conditions using AI Anxiety as Covariate

**Supplementary Material 7.** Univariate effects of AI anxiety on Feedback Reactions

**Supplementary Material 1.** Step-by-Step Participant Counts and Exclusions Across Experimental Conditions

| Sample selection stage | H | AI | AIxH | HxAI |
| --- | --- | --- | --- | --- |
| Initial sample | 53 | 66 | 51 | 60 |
| After consent | 53 | 66 | 51 | 60 |
| After attention check | 47 | 61 | 47 | 55 |
| Final sample (employed participants only) | 42 | 56 | 44 | 50 |

*Note***.** H = fully human-written, AI = fully AI-generated, AixH = AI-generated refined by a human, and HxAI = human-written refined by AI. Qualtrics was set to present the four experimental conditions evenly. Its randomizer operates using internal display counts that include incomplete questionnaires. Because the initial sample comprised only completed responses, condition sizes were not identical.

**Supplementary Material 2.** Vignettes Used in the Study

| Please read the following instructions carefully!  In the following task, we ask you to imagine yourself in a hypothetical workplace situation in which your supervisor will be a fictional character. Your task is to answer a number of questions about this situation.  You will find the description of the scenario and the related questions on the next page. | | | |
| --- | --- | --- | --- |
| *Fully human-written (H)*  It is well known that your supervisor personally writes all feedback about their subordinates. | *Fully AI-generated (AI)*  It is well known that your supervisor uses artificial intelligence to write feedback about their subordinates, which they then forward without any modification. | *AI-generated refined by human (AIxH)*  It is well known that your supervisor uses artificial intelligence to write feedback about their subordinates, which he or she personally reviews and approves before sending. | *Human-written refined by AI (HxAI)*  It is well known that your supervisor personally writes feedback about their subordinates, which they then fine-tune and refine with the help of artificial intelligence. |
| The following excerpt can be found in your most recent performance review:  „In recent months, you have been performing your work reliably and precisely. You are particularly strong in meeting deadlines and completing administrative tasks, which has greatly contributed to the team's smooth functioning. However, it would be worth paying more conscious attention to joint discussions and communication situations, especially when several perspectives need to be coordinated.” | | | |

**Supplementary Material 3.** Differences Between Conditions

*Estimated marginal means*

| Outcome | Source | *EMM* | *SE* | *df* | lower CL | upper CL |
| --- | --- | --- | --- | --- | --- | --- |
| *General affective response* | | | | | | |
|  | AI | 3.86 | 0.159 | 188 | 3.54 | 4.17 |
|  | AIxH | 4.43 | 0.179 | 188 | 4.08 | 4.79 |
|  | H | 4.76 | 0.183 | 188 | 4.40 | 5.12 |
|  | HxAI | 4.48 | 0.168 | 188 | 4.15 | 4.81 |
| *Rumination* |  |  |  |  |  |  |
|  | AI | 3.45 | 0.211 | 188 | 3.03 | 3.86 |
|  | AIxH | 3.20 | 0.238 | 188 | 2.74 | 3.67 |
|  | H | 3.64 | 0.243 | 188 | 3.16 | 4.12 |
|  | HxAI | 3.48 | 0.223 | 188 | 3.04 | 3.92 |
| *Willingness to correct the mistake* | | | | | | |
|  | AI | 4.43 | 0.197 | 188 | 4.04 | 4.82 |
|  | AIxH | 5.18 | 0.222 | 188 | 4.74 | 5.62 |
|  | H | 6.02 | 0.227 | 188 | 5.58 | 6.47 |
|  | HxAI | 5.56 | 0.208 | 188 | 5.15 | 5.97 |
| *Openness to ask for help from the supervisor* | | | | | | |
|  | AI | 3.43 | 0.209 | 188 | 3.02 | 3.84 |
|  | AIxH | 4.57 | 0.236 | 188 | 4.10 | 5.03 |
|  | H | 5.31 | 0.242 | 188 | 4.83 | 5.79 |
|  | HxAI | 4.82 | 0.221 | 188 | 4.38 | 5.26 |
| *Organizational commitment* | | | | | | |
|  | AI | 3.46 | 0.136 | 188 | 3.20 | 3.73 |
|  | AIxH | 4.00 | 0.153 | 188 | 3.70 | 4.30 |
|  | H | 4.57 | 0.157 | 188 | 4.26 | 4.88 |
|  | HxAI | 4.38 | 0.144 | 188 | 4.10 | 4.66 |
| *Relationship with supervisor* | | | | | | |
|  | AI | 3.23 | 0.140 | 188 | 2.96 | 3.51 |
|  | AIxH | 3.98 | 0.158 | 188 | 3.67 | 4.29 |
|  | H | 4.69 | 0.162 | 188 | 4.37 | 5.01 |
|  | HxAI | 4.22 | 0.148 | 188 | 3.93 | 4.51 |
| *Expected performance* | | | | | | |
|  | AI | 4.29 | 0.135 | 188 | 4.02 | 4.55 |
|  | AIxH | 4.68 | 0.152 | 188 | 4.38 | 4.98 |
|  | H | 5.19 | 0.155 | 188 | 4.88 | 5.50 |
|  | HxAI | 4.82 | 0.142 | 188 | 4.54 | 5.10 |
| *Work motivation* | | | | | | |
|  | AI | 3.66 | 0.159 | 188 | 3.35 | 3.97 |
|  | AIxH | 4.30 | 0.179 | 188 | 3.94 | 4.65 |
|  | H | 4.79 | 0.183 | 188 | 4.42 | 5.15 |
|  | HxAI | 4.68 | 0.168 | 188 | 4.35 | 5.01 |
| *Psychological distance* | | | | | | |
|  | AI | 1.67 | 0.109 | 188 | 1.45 | 1.89 |
|  | AIxH | 2.15 | 0.123 | 188 | 1.91 | 2.40 |
|  | H | 2.89 | 0.126 | 188 | 2.64 | 3.14 |
|  | HxAI | 2.54 | 0.116 | 188 | 2.31 | 2.77 |

*Note*. Confidence level used: .95.

*Bonferroni-adjusted pairwise comparisons*

| Outcome | Contrast | Estimate | *SE* | *df* | *t* ratio | *p*-value |
| --- | --- | --- | --- | --- | --- | --- |
| *General affective response* | | | | | | |
|  | AI - AIxH | -0.5747 | 0.240 | 188 | -2.399 | .1044 |
|  | AI - H | -0.9048 | 0.243 | 188 | -3.728 | .0015 |
|  | AI - HxAI | -0.6229 | 0.231 | 188 | -2.692 | .0464 |
|  | AIxH - H | -0.3301 | 0.256 | 188 | -1.287 | 1.0000 |
|  | AIxH - HxAI | -0.0482 | 0.246 | 188 | -0.196 | 1.0000 |
|  | H - HxAI | 0.2819 | 0.249 | 188 | 1.133 | 1.0000 |
| *Rumination* | | | | | | |
|  | AI - AIxH | 0.2419 | 0.318 | 188 | 0.762 | 1.0000 |
|  | AI - H | -0.1964 | 0.322 | 188 | -0.610 | 1.0000 |
|  | AI - HxAI | -0.0336 | 0.307 | 188 | -0.109 | 1.0000 |
|  | AIxH - H | -0.4383 | 0.340 | 188 | -1.289 | 1.0000 |
|  | AIxH - HxAI | -0.2755 | 0.326 | 188 | -0.845 | 1.0000 |
|  | H - HxAI | 0.1629 | 0.330 | 188 | 0.494 | 1.0000 |
| *Willingness to correct the mistake* | | | | | | |
|  | AI - AIxH | -0.7532 | 0.297 | 188 | -2.539 | .0715 |
|  | AI - H | -1.5952 | 0.301 | 188 | -5.308 | <.0001 |
|  | AI - HxAI | -1.1314 | 0.286 | 188 | -3.949 | .0007 |
|  | AIxH - H | -0.8420 | 0.318 | 188 | -2.651 | .0523 |
|  | AIxH - HxAI | -0.3782 | 0.304 | 188 | -1.243 | 1.0000 |
|  | H - HxAI | 0.4638 | 0.308 | 188 | 1.505 | .8040 |
| *Openness to ask for help from the supervisor* | | | | | | |
|  | AI - AIxH | -1.1396 | 0.315 | 188 | -3.613 | .0023 |
|  | AI - H | -1.8810 | 0.320 | 188 | -5.885 | <.0001 |
|  | AI - HxAI | -1.3914 | 0.305 | 188 | -4.568 | <.0001 |
|  | AIxH - H | -0.7413 | 0.338 | 188 | -2.195 | .1764 |
|  | AIxH - HxAI | -0.2518 | 0.324 | 188 | -0.778 | 1.0000 |
|  | H - HxAI | 0.4895 | 0.328 | 188 | 1.494 | .8215 |
| *Organizational commitment* | | | | | | |
|  | AI - AIxH | -0.5357 | 0.205 | 188 | -2.618 | .0574 |
|  | AI - H | -1.1071 | 0.207 | 188 | -5.339 | <.0001 |
|  | AI - HxAI | -0.9157 | 0.198 | 188 | -4.633 | <.0001 |
|  | AIxH - H | -0.5714 | 0.219 | 188 | -2.608 | .0591 |
|  | AIxH - HxAI | -0.3800 | 0.210 | 188 | -1.810 | .4316 |
|  | H - HxAI | 0.1914 | 0.213 | 188 | 0.900 | 1.0000 |
| *Relationship with supervisor* | | | | | | |
|  | AI - AIxH | -0.7451 | 0.211 | 188 | -3.529 | .0031 |
|  | AI - H | -1.4583 | 0.214 | 188 | -6.817 | <.0001 |
|  | AI - HxAI | -0.9879 | 0.204 | 188 | -4.844 | <.0001 |
|  | AIxH - H | -0.7132 | 0.226 | 188 | -3.154 | .0112 |
|  | AIxH - HxAI | -0.2427 | 0.217 | 188 | -1.120 | 1.0000 |
|  | H - HxAI | 0.4705 | 0.219 | 188 | 2.145 | .1996 |
| *Expected performance* | | | | | | |
|  | AI - AIxH | -0.3961 | 0.203 | 188 | -1.952 | .3148 |
|  | AI - H | -0.9048 | 0.206 | 188 | -4.399 | .0001 |
|  | AI - HxAI | -0.5343 | 0.196 | 188 | -2.726 | .0422 |
|  | AIxH - H | -0.5087 | 0.217 | 188 | -2.340 | .1219 |
|  | AIxH - HxAI | -0.1382 | 0.208 | 188 | -0.664 | 1.0000 |
|  | H - HxAI | 0.3705 | 0.211 | 188 | 1.757 | .4834 |
| *Work* *motivation* | | | | | | |
|  | AI - AIxH | -0.6347 | 0.239 | 188 | -2.651 | .0523 |
|  | AI - H | -1.1250 | 0.243 | 188 | -4.636 | <.0001 |
|  | AI - HxAI | -1.0193 | 0.231 | 188 | -4.407 | .0001 |
|  | AIxH - H | -0.4903 | 0.256 | 188 | -1.912 | .3445 |
|  | AIxH - HxAI | -0.3845 | 0.246 | 188 | -1.565 | .7156 |
|  | H - HxAI | 0.1057 | 0.249 | 188 | 0.425 | 1.0000 |
| *Psychological distance* | | | | | | |
|  | AI - AIxH | -0.4838 | 0.165 | 188 | -2.935 | .0225 |
|  | AI - H | -1.2173 | 0.167 | 188 | -7.288 | <.0001 |
|  | AI - HxAI | -0.8704 | 0.159 | 188 | -5.467 | <.0001 |
|  | AIxH - H | -0.7335 | 0.177 | 188 | -4.156 | .0003 |
|  | AIxH - HxAI | -0.3866 | 0.169 | 188 | -2.286 | .1403 |
|  | H - HxAI | 0.3469 | 0.171 | 188 | 2.026 | .2653 |

*Note.* P values were adjusted using the Bonferroni correction.

**Supplementary Material 4.** Differences Between Conditions controlling for education

The effects remained substantively unchanged when controlling for education. Education itself showed no significant multivariate effect, V = .09, F(18, 358) = 0.90, p = .584.

*Estimated marginal means*

| Outcome | Source | *EMM* | *SE* | *df* | lower CL | upper CL |
| --- | --- | --- | --- | --- | --- | --- |
| *General affective response* | | | | | | |
|  | AI | 3.70 | 0.261 | 186 | 3.18 | 4.21 |
|  | AIxH | 4.27 | 0.274 | 186 | 3.73 | 4.81 |
|  | H | 4.60 | 0.277 | 186 | 4.05 | 5.14 |
|  | HxAI | 4.38 | 0.233 | 186 | 3.92 | 4.84 |
| *Rumination* |  |  |  |  |  |  |
|  | AI | 3.61 | 0.345 | 186 | 2.93 | 4.29 |
|  | AIxH | 3.36 | 0.362 | 186 | 2.64 | 4.07 |
|  | H | 3.81 | 0.366 | 186 | 3.09 | 4.53 |
|  | HxAI | 3.55 | 0.308 | 186 | 2.94 | 4.16 |
| *Willingness to correct the mistake* | | | | | | |
|  | AI | 4.43 | 0.323 | 186 | 3.79 | 5.07 |
|  | AIxH | 5.19 | 0.340 | 186 | 4.52 | 5.86 |
|  | H | 6.02 | 0.343 | 186 | 5.34 | 6.69 |
|  | HxAI | 5.60 | 0.288 | 186 | 5.03 | 6.17 |
| *Openness to ask for help from the supervisor* | | | | | | |
|  | AI | 3.70 | 0.343 | 186 | 3.02 | 4.37 |
|  | AIxH | 4.84 | 0.360 | 186 | 4.13 | 5.56 |
|  | H | 5.57 | 0.363 | 186 | 4.86 | 6.29 |
|  | HxAI | 5.07 | 0.306 | 186 | 4.47 | 5.68 |
| *Organizational commitment* | | | | | | |
|  | AI | 3.51 | 0.224 | 186 | 3.07 | 3.95 |
|  | AIxH | 4.05 | 0.235 | 186 | 3.58 | 4.51 |
|  | H | 4.62 | 0.237 | 186 | 4.15 | 5.09 |
|  | HxAI | 4.42 | 0.200 | 186 | 4.03 | 4.82 |
| *Relationship with supervisor* | | | | | | |
|  | AI | 3.16 | 0.231 | 186 | 2.71 | 3.62 |
|  | AIxH | 3.91 | 0.242 | 186 | 3.43 | 4.39 |
|  | H | 4.62 | 0.244 | 186 | 4.14 | 5.10 |
|  | HxAI | 4.19 | 0.206 | 186 | 3.78 | 4.59 |
| *Expected performance* | | | | | | |
|  | AI | 4.28 | 0.219 | 186 | 3.85 | 4.72 |
|  | AIxH | 4.69 | 0.230 | 186 | 4.23 | 5.14 |
|  | H | 5.18 | 0.232 | 186 | 4.72 | 5.64 |
|  | HxAI | 4.87 | 0.196 | 186 | 4.48 | 5.25 |
| *Work motivation* | | | | | | |
|  | AI | 3.62 | 0.259 | 186 | 3.11 | 4.13 |
|  | AIxH | 4.26 | 0.272 | 186 | 3.73 | 4.80 |
|  | H | 4.74 | 0.275 | 186 | 4.20 | 5.28 |
|  | HxAI | 4.70 | 0.231 | 186 | 4.25 | 5.16 |
| *Psychological distance* | | | | | | |
|  | AI | 1.62 | 0.180 | 186 | 1.26 | 1.97 |
|  | AIxH | 2.10 | 0.189 | 186 | 1.73 | 2.47 |
|  | H | 2.83 | 0.191 | 186 | 2.45 | 3.21 |
|  | HxAI | 2.51 | 0.161 | 186 | 2.19 | 2.83 |

*Note*. Results are averaged over the levels of education. Confidence level used: .95.

*Bonferroni-adjusted pairwise comparisons*

| Outcome | Contrast | Estimate | *SE* | *df* | *t* ratio | *p*-value |
| --- | --- | --- | --- | --- | --- | --- |
| *General affective response* | | | | | | |
|  | AI - AIxH | -0.578 | 0.240 | 186 | -2.412 | .1010 |
|  | AI - H | -0.902 | 0.243 | 186 | -3.711 | .0016 |
|  | AI - HxAI | -0.686 | 0.238 | 186 | -2.884 | .0264 |
|  | AIxH - H | -0.323 | 0.257 | 186 | -1.258 | 1.0000 |
|  | AIxH - HxAI | -0.108 | 0.252 | 186 | -0.428 | 1.0000 |
|  | H - HxAI | 0.215 | 0.255 | 186 | 0.843 | 1.0000 |
| *Rumination* | | | | | | |
|  | AI - AIxH | 0.2501 | 0.317 | 186 | 0.789 | 1.0000 |
|  | AI - H | -0.2034 | 0.321 | 186 | -0.633 | 1.0000 |
|  | AI - HxAI | 0.0589 | 0.314 | 186 | 0.187 | 1.0000 |
|  | AIxH - H | -0.4534 | 0.339 | 186 | -1.336 | 1.0000 |
|  | AIxH - HxAI | -0.1912 | 0.333 | 186 | -0.575 | 1.0000 |
|  | H - HxAI | 0.2622 | 0.337 | 186 | 0.777 | 1.0000 |
| *Willingness to correct the mistake* | | | | | | |
|  | AI - AIxH | -0.760 | 0.297 | 186 | -2.560 | .0676 |
|  | AI - H | -1.590 | 0.301 | 186 | -5.286 | <.0001 |
|  | AI - HxAI | -1.175 | 0.295 | 186 | -3.988 | .0006 |
|  | AIxH - H | -0.830 | 0.318 | 186 | -2.610 | .0587 |
|  | AIxH - HxAI | -0.415 | 0.312 | 186 | -1.331 | 1.0000 |
|  | H - HxAI | 0.415 | 0.316 | 186 | 1.313 | 1.0000 |
| *Openness to ask for help from the supervisor* | | | | | | |
|  | AI - AIxH | -1.147 | 0.315 | 186 | -3.643 | .0021 |
|  | AI - H | -1.875 | 0.319 | 186 | -5.877 | <.0001 |
|  | AI - HxAI | -1.374 | 0.312 | 186 | -4.399 | .0001 |
|  | AIxH - H | -0.728 | 0.337 | 186 | -2.159 | .1930 |
|  | AIxH - HxAI | -0.227 | 0.330 | 186 | -0.688 | 1.0000 |
|  | H - HxAI | 0.500 | 0.335 | 186 | 1.493 | .8235 |
| *Organizational commitment* | | | | | | |
|  | AI - AIxH | -0.537 | 0.206 | 186 | -2.609 | .0588 |
|  | AI - H | -1.106 | 0.208 | 186 | -5.308 | <.0001 |
|  | AI - HxAI | -0.911 | 0.204 | 186 | -4.464 | .0001 |
|  | AIxH - H | -0.570 | 0.220 | 186 | -2.585 | .0630 |
|  | AIxH - HxAI | -0.374 | 0.216 | 186 | -1.734 | .5070 |
|  | H - HxAI | 0.195 | 0.219 | 186 | 0.891 | 1.0000 |
| *Relationship with supervisor* | | | | | | |
|  | AI - AIxH | -0.748 | 0.212 | 186 | -3.535 | .0031 |
|  | AI - H | -1.456 | 0.214 | 186 | -6.786 | <.0001 |
|  | AI - HxAI | -1.026 | 0.210 | 186 | -4.884 | <.0001 |
|  | AIxH - H | -0.707 | 0.227 | 186 | -3.118 | .0126 |
|  | AIxH - HxAI | -0.278 | 0.222 | 186 | -1.249 | 1.0000 |
|  | H - HxAI | 0.430 | 0.225 | 186 | 1.905 | .3496 |
| *Expected performance* | | | | | | |
|  | AI - AIxH | -0.404 | 0.201 | 186 | -2.003 | .2797 |
|  | AI - H | -0.898 | 0.204 | 186 | -4.402 | .0001 |
|  | AI - HxAI | -0.583 | 0.200 | 186 | -2.918 | .0238 |
|  | AIxH - H | -0.495 | 0.216 | 186 | -2.294 | .1375 |
|  | AIxH - HxAI | -0.180 | 0.211 | 186 | -0.850 | 1.0000 |
|  | H - HxAI | 0.315 | 0.215 | 186 | 1.470 | .8602 |
| *Work motivation* | | | | | | |
|  | AI - AIxH | -0.6430 | 0.238 | 186 | -2.703 | .0451 |
|  | AI - H | -1.1180 | 0.241 | 186 | -4.637 | <.0001 |
|  | AI - HxAI | -1.0829 | 0.236 | 186 | -4.587 | <.0001 |
|  | AIxH - H | -0.4749 | 0.255 | 186 | -1.863 | .3838 |
|  | AIxH - HxAI | -0.4399 | 0.250 | 186 | -1.761 | .4791 |
|  | H - HxAI | 0.0351 | 0.253 | 186 | 0.138 | 1.0000 |
| *Psychological distance* | | | | | | |
|  | AI - AIxH | -0.486 | 0.165 | 186 | -2.934 | .0226 |
|  | AI - H | -1.216 | 0.168 | 186 | -7.251 | <.0001 |
|  | AI - HxAI | -0.895 | 0.164 | 186 | -5.449 | <.0001 |
|  | AIxH - H | -0.730 | 0.177 | 186 | -4.120 | .0003 |
|  | AIxH - HxAI | -0.409 | 0.174 | 186 | -2.356 | .1169 |
|  | H - HxAI | 0.321 | 0.176 | 186 | 1.821 | .4211 |

*Note*. Results are averaged over the levels of education. P values were adjusted using the Bonferroni correction.

**Supplementary Material 5.** Robustness Analysis Among Participants Who Passed the Manipulation Check

*Univariate effects of feedback source*

| Outcome | *F*(3, 119) | p | Partial *η*² |
| --- | --- | --- | --- |
| General affective response | 9.42 | < .001 | .19 |
| Rumination | 0.08 | .970 | .002 |
| Willingness to correct the mistake | 14.82 | < .001 | .27 |
| Openness to ask for help | 19.23 | < .001 | .33 |
| Organizational commitment | 15.41 | < .001 | .28 |
| Relationship with supervisor | 26.56 | < .001 | .40 |
| Expected performance | 12.11 | < .001 | .23 |
| Work motivation | 14.77 | < .001 | .27 |
| Psychological distance | 28.89 | < .001 | .42 |

*Estimated marginal means*

| Outcome | Source | *EMM* | *SE* | *df* | Lower CL | Upper CL |
| --- | --- | --- | --- | --- | --- | --- |
| ***General affective response*** | | | | | | |
|  | H | 5.07 | 0.207 | 119 | 4.66 | 5.48 |
|  | AI | 3.63 | 0.189 | 119 | 3.25 | 4.00 |
|  | AIxH | 4.42 | 0.219 | 119 | 3.99 | 4.86 |
|  | HxAI | 4.61 | 0.194 | 119 | 4.22 | 4.99 |
| ***Rumination*** |  |  |  |  |  |  |
|  | H | 3.45 | 0.303 | 119 | 2.85 | 4.05 |
|  | AI | 3.37 | 0.276 | 119 | 2.82 | 3.92 |
|  | AIxH | 3.58 | 0.320 | 119 | 2.94 | 4.21 |
|  | HxAI | 3.42 | 0.284 | 119 | 2.86 | 3.99 |
| ***Willingness to correct the mistake*** | | | | | | |
|  | H | 6.10 | 0.232 | 119 | 5.64 | 6.56 |
|  | AI | 4.17 | 0.211 | 119 | 3.75 | 4.59 |
|  | AIxH | 5.46 | 0.245 | 119 | 4.98 | 5.95 |
|  | HxAI | 5.70 | 0.217 | 119 | 5.27 | 6.13 |
| ***Openness to ask for help*** | | | | | | |
|  | H | 5.66 | 0.262 | 119 | 5.14 | 6.17 |
|  | AI | 3.11 | 0.239 | 119 | 2.64 | 3.59 |
|  | AIxH | 4.54 | 0.277 | 119 | 3.99 | 5.09 |
|  | HxAI | 5.03 | 0.246 | 119 | 4.54 | 5.52 |
| ***Organizational commitment*** | | | | | | |
|  | H | 4.66 | 0.180 | 119 | 4.30 | 5.01 |
|  | AI | 3.26 | 0.163 | 119 | 2.93 | 3.58 |
|  | AIxH | 3.81 | 0.190 | 119 | 3.43 | 4.18 |
|  | HxAI | 4.58 | 0.168 | 119 | 4.24 | 4.91 |
| ***Relationship with supervisor*** | | | | | | |
|  | H | 4.97 | 0.180 | 119 | 4.61 | 5.32 |
|  | AI | 2.89 | 0.164 | 119 | 2.56 | 3.21 |
|  | AIxH | 3.81 | 0.190 | 119 | 3.43 | 4.18 |
|  | HxAI | 4.33 | 0.169 | 119 | 4.00 | 4.67 |
| ***Expected performance*** | | | | | | |
|  | H | 5.38 | 0.173 | 119 | 5.04 | 5.72 |
|  | AI | 4.00 | 0.158 | 119 | 3.69 | 4.31 |
|  | AIxH | 4.58 | 0.183 | 119 | 4.21 | 4.94 |
|  | HxAI | 4.85 | 0.162 | 119 | 4.53 | 5.17 |
| ***Work motivation*** | | | | | | |
|  | H | 4.86 | 0.204 | 119 | 4.46 | 5.27 |
|  | AI | 3.31 | 0.185 | 119 | 2.95 | 3.68 |
|  | AIxH | 4.19 | 0.215 | 119 | 3.77 | 4.62 |
|  | HxAI | 4.85 | 0.191 | 119 | 4.47 | 5.23 |
| ***Psychological distance*** | | | | | | |
|  | H | 3.12 | 0.141 | 119 | 2.84 | 3.40 |
|  | AI | 1.44 | 0.128 | 119 | 1.19 | 1.70 |
|  | AIxH | 2.06 | 0.149 | 119 | 1.76 | 2.35 |
|  | HxAI | 2.61 | 0.132 | 119 | 2.35 | 2.88 |

*Note*. Confidence level used: .95.

*Bonferroni-adjusted pairwise comparisons*

| Outcome | Contrast | Estimate | *SE* | *df* | t ratio | p-value |  |
| --- | --- | --- | --- | --- | --- | --- | --- |
| ***General affective response*** | | | | | | | |
|  | H − AI | 1.4404 | 0.280 | 119 | 5.139 | < .0001 |  |
|  | H − AIxH | 0.6459 | 0.301 | 119 | 2.143 | .2051 |  |
|  | H − HxAI | 0.4629 | 0.284 | 119 | 1.629 | .6353 |  |
|  | AI − AIxH | −0.7945 | 0.289 | 119 | −2.749 | .0414 |  |
|  | AI − HxAI | −0.9775 | 0.271 | 119 | −3.609 | .0027 |  |
|  | AIxH − HxAI | −0.1830 | 0.293 | 119 | −0.625 | 1.0000 |  |
| ***Rumination*** | | | | | | | |
|  | H − AI | 0.0768 | 0.410 | 119 | 0.187 | 1.0000 |  |
|  | H − AIxH | −0.1286 | 0.441 | 119 | −0.291 | 1.0000 |  |
|  | H − HxAI | 0.0240 | 0.416 | 119 | 0.058 | 1.0000 |  |
|  | AI − AIxH | −0.2055 | 0.423 | 119 | −0.486 | 1.0000 |  |
|  | AI − HxAI | −0.0528 | 0.396 | 119 | −0.133 | 1.0000 |  |
|  | AIxH − HxAI | 0.1527 | 0.429 | 119 | 0.356 | 1.0000 |  |
| ***Willingness to correct the mistake*** | | | | | | | |
|  | H − AI | 1.9320 | 0.313 | 119 | 6.169 | < .0001 |  |
|  | H − AIxH | 0.6419 | 0.337 | 119 | 1.906 | .3546 |  |
|  | H − HxAI | 0.4065 | 0.317 | 119 | 1.280 | 1.0000 |  |
|  | AI − AIxH | −1.2901 | 0.323 | 119 | −3.995 | .0007 |  |
|  | AI − HxAI | −1.5255 | 0.303 | 119 | −5.041 | < .0001 |  |
|  | AIxH − HxAI | −0.2354 | 0.327 | 119 | −0.720 | 1.0000 |  |
| ***Openness to ask for help*** | | | | | | | |
|  | H − AI | 2.5409 | 0.355 | 119 | 7.162 | < .0001 |  |
|  | H − AIxH | 1.1167 | 0.382 | 119 | 2.927 | .0246 |  |
|  | H − HxAI | 0.6249 | 0.360 | 119 | 1.738 | .5091 |  |
|  | AI − AIxH | −1.4242 | 0.366 | 119 | −3.893 | < .001 |  |
|  | AI − HxAI | −1.9160 | 0.343 | 119 | −5.589 | < .0001 |  |
|  | AIxH − HxAI | −0.4918 | 0.370 | 119 | −1.328 | 1.0000 |  |
| ***Organizational commitment*** | | | | | | | |
|  | H − AI | 1.3980 | 0.243 | 119 | 5.756 | < .0001 |  |
|  | H − AIxH | 0.8475 | 0.261 | 119 | 3.244 | .0092 |  |
|  | H − HxAI | 0.0794 | 0.246 | 119 | 0.323 | 1.0000 |  |
|  | AI − AIxH | −0.5505 | 0.250 | 119 | −2.198 | .1791 |  |
|  | AI − HxAI | −1.3186 | 0.235 | 119 | −5.618 | < .0001 |  |
|  | AIxH − HxAI | −0.7681 | 0.254 | 119 | −3.028 | .0181 |  |
| ***Relationship with supervisor*** | | | | | | | |
|  | H − AI | 2.0798 | 0.243 | 119 | 8.542 | < .0001 |  |
|  | H − AIxH | 1.1578 | 0.262 | 119 | 4.421 | .0001 |  |
|  | H − HxAI | 0.6322 | 0.247 | 119 | 2.562 | .0700 |  |
|  | AI − AIxH | −0.9220 | 0.251 | 119 | −3.673 | .0022 |  |
|  | AI − HxAI | −1.4476 | 0.235 | 119 | −6.153 | < .0001 |  |
|  | AIxH − HxAI | −0.5256 | 0.254 | 119 | −2.067 | .2452 |  |
| ***Expected performance*** | | | | | | | |
|  | H − AI | 1.3793 | 0.234 | 119 | 5.892 | < .0001 |  |
|  | H − AIxH | 0.8024 | 0.252 | 119 | 3.187 | .0110 |  |
|  | H − HxAI | 0.5308 | 0.237 | 119 | 2.237 | .1628 |  |
|  | AI − AIxH | −0.5769 | 0.241 | 119 | −2.390 | .1104 |  |
|  | AI − HxAI | −0.8485 | 0.226 | 119 | −3.751 | .0016 |  |
|  | AIxH − HxAI | −0.2716 | 0.244 | 119 | −1.111 | 1.0000 |  |
| ***Work motivation*** | | | | | | | |
|  | H − AI | 1.5478 | 0.276 | 119 | 5.618 | < .0001 |  |
|  | H − AIxH | 0.6698 | 0.296 | 119 | 2.260 | .1538 |  |
|  | H − HxAI | 0.0136 | 0.279 | 119 | 0.049 | 1.0000 |  |
|  | AI − AIxH | −0.8780 | 0.284 | 119 | −3.091 | .0149 |  |
|  | AI − HxAI | −1.5342 | 0.266 | 119 | −5.763 | < .0001 |  |
|  | AIxH − HxAI | −0.6562 | 0.288 | 119 | −2.281 | .1461 |  |
| **Psychological distance** | | | | | | | |
|  | H − AI | 1.6778 | 0.191 | 119 | 8.798 | < .0001 |  |
|  | H − AIxH | 1.0630 | 0.205 | 119 | 5.182 | < .0001 |  |
|  | H − HxAI | 0.5071 | 0.193 | 119 | 2.623 | .0591 |  |
|  | AI − AIxH | −0.6148 | 0.197 | 119 | −3.127 | .0133 |  |
|  | AI − HxAI | −1.1708 | 0.184 | 119 | −6.353 | < .0001 |  |
|  | AIxH − HxAI | −0.5559 | 0.199 | 119 | −2.792 | .0367 |  |

*Note*. P values were adjusted using the Bonferroni correction.

**Supplementary Material 6.** Differences Between Conditions using AI Anxiety as Covariate

*Estimated marginal means*

| Outcome | Source | *EMM* | *SE* | *df* | lower CL | upper CL |
| --- | --- | --- | --- | --- | --- | --- |
| *General affective response* | | | | | | |
|  | AI | 3.86 | 0.159 | 187 | 3.55 | 4.17 |
|  | AIxH | 4.42 | 0.180 | 187 | 4.07 | 4.78 |
|  | H | 4.76 | 0.184 | 187 | 4.40 | 5.12 |
|  | HxAI | 4.49 | 0.169 | 187 | 4.16 | 4.82 |
| *Rumination* |  |  |  |  |  |  |
|  | AI | 3.44 | 0.203 | 187 | 3.03 | 3.84 |
|  | AIxH | 3.27 | 0.230 | 187 | 2.81 | 3.72 |
|  | H | 3.65 | 0.235 | 187 | 3.19 | 4.12 |
|  | HxAI | 3.43 | 0.216 | 187 | 3.00 | 3.86 |
| *Willingness to correct the mistake* | | | | | | |
|  | AI | 4.43 | 0.197 | 187 | 4.04 | 4.82 |
|  | AIxH | 5.19 | 0.223 | 187 | 4.75 | 5.63 |
|  | H | 6.03 | 0.228 | 187 | 5.58 | 6.47 |
|  | HxAI | 5.55 | 0.209 | 187 | 5.14 | 5.96 |
| *Openness to ask for help from the supervisor* | | | | | | |
|  | AI | 3.43 | 0.210 | 187 | 3.02 | 3.84 |
|  | AIxH | 4.57 | 0.237 | 187 | 4.10 | 5.03 |
|  | H | 5.31 | 0.242 | 187 | 4.83 | 5.79 |
|  | HxAI | 4.82 | 0.222 | 187 | 4.38 | 5.26 |
| *Organizational commitment* | | | | | | |
|  | AI | 3.47 | 0.136 | 187 | 3.20 | 3.73 |
|  | AIxH | 3.99 | 0.154 | 187 | 3.69 | 4.30 |
|  | H | 4.57 | 0.157 | 187 | 4.26 | 4.88 |
|  | HxAI | 4.39 | 0.144 | 187 | 4.10 | 4.67 |
| *Relationship with supervisor* | | | | | | |
|  | AI | 3.23 | 0.140 | 187 | 2.96 | 3.51 |
|  | AIxH | 3.97 | 0.158 | 187 | 3.65 | 4.28 |
|  | H | 4.69 | 0.162 | 187 | 4.37 | 5.01 |
|  | HxAI | 4.23 | 0.148 | 187 | 3.94 | 4.52 |
| *Expected performance* | | | | | | |
|  | AI | 4.29 | 0.135 | 187 | 4.02 | 4.55 |
|  | AIxH | 4.68 | 0.153 | 187 | 4.38 | 4.98 |
|  | H | 5.19 | 0.156 | 187 | 4.88 | 5.50 |
|  | HxAI | 4.82 | 0.143 | 187 | 4.54 | 5.10 |
| *Work motivation* | | | | | | |
|  | AI | 3.66 | 0.159 | 187 | 3.35 | 3.98 |
|  | AIxH | 4.29 | 0.180 | 187 | 3.93 | 4.64 |
|  | H | 4.78 | 0.184 | 187 | 4.42 | 5.15 |
|  | HxAI | 4.69 | 0.169 | 187 | 4.35 | 5.02 |
| *Psychological distance* | | | | | | |
|  | AI | 2.00 | 0.164 | 187 | 1.68 | 2.33 |
|  | AIxH | 2.73 | 0.186 | 187 | 2.37 | 3.10 |
|  | H | 3.83 | 0.190 | 187 | 3.46 | 4.21 |
|  | HxAI | 3.31 | 0.174 | 187 | 2.96 | 3.65 |

*Note*. Results are averaged over the levels of education. Confidence level used: .95.

*Bonferroni-adjusted pairwise comparisons*

| Outcome | Contrast | Estimate | *SE* | *df* | *t* ratio | *p*-value |
| --- | --- | --- | --- | --- | --- | --- |
| *General affective response* | | | | | | |
|  | AI - AIxH | -0.562 | 0.240 | 187 | -2.341 | .1218 |
|  | AI - H | -0.901 | 0.243 | 187 | -3.710 | .0016 |
|  | AI - HxAI | -0.630 | 0.232 | 187 | -2.719 | .0430 |
|  | AIxH - H | -0.339 | 0.257 | 187 | -1.320 | 1.0000 |
|  | AIxH - HxAI | -0.068 | 0.247 | 187 | -0.274 | 1.0000 |
|  | H - HxAI | 0.271 | 0.249 | 187 | 1.089 | 1.0000 |
| *Rumination* | | | | | | |
|  | AI - AIxH | 0.170 | 0.307 | 187 | 0.555 | 1.0000 |
|  | AI - H | -0.217 | 0.311 | 187 | -0.699 | 1.0000 |
|  | AI - HxAI | 0.006 | 0.296 | 187 | 0.019 | 1.0000 |
|  | AIxH - H | -0.387 | 0.329 | 187 | -1.179 | 1.0000 |
|  | AIxH - HxAI | -0.165 | 0.316 | 187 | -0.521 | 1.0000 |
|  | H - HxAI | 0.223 | 0.319 | 187 | 0.698 | 1.0000 |
| *Willingness to correct the mistake* | | | | | | |
|  | AI - AIxH | -0.765 | 0.298 | 187 | -2.569 | .0659 |
|  | AI - H | -1.598 | 0.301 | 187 | -5.309 | <.0001 |
|  | AI - HxAI | -1.125 | 0.287 | 187 | -3.919 | .0007 |
|  | AIxH - H | -0.834 | 0.318 | 187 | -2.619 | .0572 |
|  | AIxH - HxAI | -0.361 | 0.306 | 187 | -1.178 | 1.0000 |
|  | H - HxAI | 0.473 | 0.309 | 187 | 1.531 | .7643 |
| *Openness to ask for help from the supervisor* | | | | | | |
|  | AI - AIxH | -1.137 | 0.317 | 187 | -3.587 | .0026 |
|  | AI - H | -1.880 | 0.320 | 187 | -5.866 | <.0001 |
|  | AI - HxAI | -1.393 | 0.306 | 187 | -4.558 | <.0001 |
|  | AIxH - H | -0.744 | 0.339 | 187 | -2.194 | .1769 |
|  | AIxH - HxAI | -0.257 | 0.326 | 187 | -0.787 | 1.0000 |
|  | H - HxAI | 0.487 | 0.329 | 187 | 1.480 | .8428 |
| *Organizational commitment* | | | | | | |
|  | AI - AIxH | -0.528 | 0.205 | 187 | -2.569 | .0658 |
|  | AI - H | -1.105 | 0.208 | 187 | -5.319 | <.0001 |
|  | AI - HxAI | -0.920 | 0.198 | 187 | -4.646 | <.0001 |
|  | AIxH - H | -0.577 | 0.220 | 187 | -2.628 | .0558 |
|  | AIxH - HxAI | -0.393 | 0.211 | 187 | -1.860 | .3871 |
|  | H - HxAI | 0.185 | 0.213 | 187 | 0.866 | 1.0000 |
| *Relationship with supervisor* | | | | | | |
|  | AI - AIxH | -0.732 | 0.211 | 187 | -3.460 | .0040 |
|  | AI - H | -1.455 | 0.214 | 187 | -6.799 | <.0001 |
|  | AI - HxAI | -0.995 | 0.204 | 187 | -4.878 | <.0001 |
|  | AIxH - H | -0.723 | 0.226 | 187 | -3.194 | .0099 |
|  | AIxH - HxAI | -0.263 | 0.218 | 187 | -1.211 | 1.0000 |
|  | H - HxAI | 0.459 | 0.220 | 187 | 2.092 | .2268 |
| *Expected performance* | | | | | | |
|  | AI - AIxH | -0.394 | 0.204 | 187 | -1.930 | .3305 |
|  | AI - H | -0.904 | 0.206 | 187 | -4.384 | .0001 |
|  | AI - HxAI | -0.536 | 0.197 | 187 | -2.724 | .0423 |
|  | AIxH - H | -0.511 | 0.218 | 187 | -2.341 | .1217 |
|  | AIxH - HxAI | -0.142 | 0.210 | 187 | -0.678 | 1.0000 |
|  | H - HxAI | 0.368 | 0.212 | 187 | 1.740 | .5010 |
| *Work motivation* | | | | | | |
|  | AI - AIxH | -0.627 | 0.240 | 187 | -2.607 | .0591 |
|  | AI - H | -1.123 | 0.243 | 187 | -4.618 | <.0001 |
|  | AI - HxAI | -1.024 | 0.232 | 187 | -4.415 | .0001 |
|  | AIxH - H | -0.496 | 0.257 | 187 | -1.929 | .3316 |
|  | AIxH - HxAI | -0.397 | 0.247 | 187 | -1.606 | .6604 |
|  | H - HxAI | 0.099 | 0.250 | 187 | 0.397 | 1.0000 |
| *Psychological distance* | | | | | | |
|  | AI - AIxH | -0.730 | 0.248 | 187 | -2.940 | .0222 |
|  | AI - H | -1.827 | 0.251 | 187 | -7.274 | <.0001 |
|  | AI - HxAI | -1.303 | 0.240 | 187 | -5.441 | <.0001 |
|  | AIxH - H | -1.097 | 0.266 | 187 | -4.130 | .0003 |
|  | AIxH - HxAI | -0.573 | 0.255 | 187 | -2.244 | .1560 |
|  | H - HxAI | 0.524 | 0.258 | 187 | 2.032 | .2611 |

*Note*. Results are averaged over the levels of education. P values were adjusted using the Bonferroni correction.

**Supplementary Material 7.** Univariate effects of AI anxiety on Feedback Reactions

| Outcome | *F*(1,187) | *p* | Partial η² |
| --- | --- | --- | --- |
| General affective response | 0.75 | .389 | .004 |
| Rumination | 14.76 | <.001 | .073 |
| Willingness to correct the mistake | 0.39 | .532 | .002 |
| Openness to ask for help | 0.03 | .873 | <.001 |
| Organizational commitment | 0.43 | .512 | .002 |
| Relationship with supervisor | 1.08 | .301 | .006 |
| Expected performance | 0.04 | .834 | <.001 |
| Work motivation | 0.30 | .585 | .002 |
| Psychological distance | 0.08 | .772 | <.001 |
